# *Quasipucciniastrum agrimoniae*, gen. et sp. nov. on *Agrimonia* (Rosaceae) from China

**DOI:** 10.1080/21501203.2019.1610522

**Published:** 2019-04-30

**Authors:** Xiao-Hua Qi, Lei Cai, Peng Zhao

**Affiliations:** a State Key Laboratory of Mycology, Institute of Microbiology, Chinese Academy of Sciences, Beijing, China; b University of Chinese Academy of Sciences, Beijing, China

**Keywords:** Agrimony, Cronartiaceae, molecular phylogeny, morphology, Pucciniastraceae

## Abstract

A new rust genus, *Quasipucciniastrum*, typified by *Q. agrimoniae* sp. nov., is proposed based on distinct morphological characters and phylogenetic placement. This genus is characterised by its uredinial ostiolar peridial cells with rough surface and sessile, multicellular teliospores with apparently thickened apical wall. Molecular phylogenetic analyses using internal transcribed spacer regions and intervening 5.8S nrRNA gene (ITS) and the large subunit (LSU) rDNA revealed that this genus is sister to the genus *Cronartium* (Cronartiaceae), but morphologically it is distinct from *Cronartium* in the sessile teliospores that are divided by vertical septa. Morphologically, *Quasipucciniastrum* should be compared to *Pucciniastrum* (Pucciniastraceae) in its multicellular teliospores, but they were phylogenetically distant.

## Introduction

*Agrimonia* Ldb. species, known as “common agrimony”, are perennial herbaceous flowering plants widely distributed in the temperate regions of the Northern Hemisphere, and they have been commonly planted for ornamentation and medicinal use (Lu 2001). Common agrimony is economically and horticulturally important, but their growths have been frequently threatened by rust diseases. Hitherto, seven rust species have been recorded on *Agrimonia* species (Farr and Rossman 2018), among which, *Pucciniastrum agrimoniae* (Dietel) Tranzschel (Pucciniastraceae) and its synonymies, *P. agrimoniae-eupatoriae* (DC.) Lagerh. (Pucciniastraceae), *Thekopsora agrimoniae* Dietel (Pucciniastraceae), *Uredo agrimoniae* J. Schröt. (Anamorphic genera) are frequently assigned names (Tai 1979; Zhuang 1983, 2001, 2005; Guo 1989; Zhang et al. 1997; Cao and Li 1999; Zhuang and Wei 1999; Cao et al. 2000), or in fewer cases as *Melampsora agrimoniae* Dietel (Melampsoraceae), *Puccinia agrimoniae* (Arthur) Arthur (Pucciniaceae) and *Uropyxis agrimoniae* Arthur (Uropyxidaceae) (Arthur 1910; Maneval 1937).

During our study on rust fungi in China, a morphologically distinct species was found on *Agrimonia pilosa*. It produces *Milesia*-type uredinia and ostiolar peridial cells with rough surface, and have subglobose teliospores divided by vertical septa. These characters are to some extent, similar to *Pucciniastrum* spp. (Pucciniastraceae), but the rDNA ITS and LSU sequences showed its close relationship to genus *Cronartium* Fr. (Cronartiaceae) rather than *Pucciniastrum*. Our critical morphological and molecular comparisons of this fungus with *Cronartium, Pucciniastrum* and other related genera suggested that this rust fungus represents a new genus herein described as *Quasipucciniastrum agrimoniae* gen. et sp. nov.

## Materials and methods

### Fungal specimens

A total of 204 dried herbarium specimens on *Agrimonia pilosa* were loaned from the Mycological Herbarium of Institute of Microbiology, CAS, China (HMAS). Several fresh specimens on *Agrimonia* species were collected from different provinces in China during last three years. Herbarium specimens of *Cronartium* and *Peridermium* (Link) J.C. Schmidt & Kunze involved in this study were loaned from University of Florida Herbarium, USA (FLAS), HMAS, University of Michigan Herbarium, USA (MICH) and New York Botanical Garden, USA (NY). In this study, the Roman numerals of II and III referred to uredinial and telial stages in the rust fungi.

### Morphological examinations

Morphological characteristics of all specimens were observed using a dissecting microscope (DM), the light microscope (LM) and the scanning electron microscope (SEM). The methods for morphological analyses as outlined by Zhao et al. (2013), Zhao et al. (2014), 2017) were followed. Fifty measurements of sori and spores from each specimen were recorded, and morphological characteristics in uredinial and telial stages, i.e. the position of uredinia and telia, the ornamentation and dimension of urediniospores, the dimension and shape of ostiolar cells, the position and shape of peridial cells, the dimension of teliospores, the position and shape of teliospores were examined.

### DNA extraction, amplification and sequencing

For the fungal specimens, several sori from each specimen were excised and DNA were extracted from all studied herbarium specimens using Gentra Puregene Tissue Kit (Qiagen, Valencia, CA) according to the manufacturer’s instructions. From the crude extracts, 1–3 μl DNA templates were directly used for the polymerase chain reaction (PCR) amplification of the internal transcribed spacer regions and intervening 5.8S nrRNA gene (ITS) and the large subunit (LSU) rDNA. rDNA ITS regions were amplified using the primer pairs ITS1F (Gardes and Bruns 1993)/ITS4 (White et al. 1990), Rust2inv (Aime 2006)/ITS4BR (Feau et al. 2009), and fragment of LSU was amplified using the primer pairs ITS4BRf/LR6 (Vilgalys and Hester 1990), LR1R/LR3 and LR3R/LR6 (Vilgalys and Hester 1990). PCR was performed under the following conditions: denaturation at 95°C for 5 min; followed by 35 cycles of denaturation at 95°C for 45 s, annealing at 55°C for 1 min and elongation at 72°C for 1 min, finally with an extension step at 72°C for 10 min. Purification and sequencing of PCR amplicons were carried out at the Tianyi Huiyuan Company, Beijing.

### Molecular phylogeny

Full-length ITS and partial LSU regions were amplified from 23 specimens, and we included 16 specimens from genus *Cronartium* and *Peridermium* for comparable studies because they have high similarity to rust fungus on *Agrimonia* in rDNA sequences. Their herbarium numbers, host plants, geographical origins and GenBank accession numbers are listed in Table 1. Besides, a total of 148 sequences from closely related species were retrieved from GenBank for phylogenetic comparison (Table 2). *Pileolaria terebinthi* (DC.) Castagne was used as outgroup. In the final dataset, ITS and LSU were concatenated and the final alignment includes 124 specimens with a length of 1836 characters (743 for ITS, 1093 for 28S).

**Table 1.** Herbarium specimens used for molecular phylogenetic analyses.

				**GenBank accession no. Collection**	
**Species**	**Host**	**Locality and data of**	**Specimen no.^a^**	**LSU**	**ITS**
*Cronartium coleosporioides*	*Castilleja linariaefolia*	Montana USA, 1914	MICH253346	MK208284	MK193824
*C. comandrae*	*Comandra umbellata*	Wisconsin USA, 1955	MICH253364	MK208293	MK193825
*C. comandrae*	*C. richardsiana*	Canda	NY00267638	MK208294	MK193826
*C. comandrae*	*C. pallida*	USA	NY03106200	MK208295	MK193827
*C. flaccidum*	*Paeonia lactiflora*	Ussuriysk Russia, 2003	HMAS89229	MK208286	MK193819
*C. flaccidum*	*P. lactiflora*	Jilin China, 2003	HMAS89231	MK208289	MK193822
*C. flaccidum*	*Pinus tabulaeformis*	Inner Mongolia China, 1984	HMAS82784	MK208287	MK193818
*C. flaccidum*	*P.taiwanensis*	Henan China, 1981	HMAS44164	MK208288	MK193816
*C. flaccidum*	*Quercus aquifolioides*	Tibet China, 2010	HMAS242642	MK208290	MK193821
*C. flaccidum*	*Q. aquifolioides*	Tibet China, 2010	HMAS242641	MK208291	MK193820
*C. quercuum*	*Q. glandulifera*	Jiangxi China, 1996	HMAS82717	MK208292	MK193817
*C. ribicola*	*Ribes nigrum*	USA	NY00267051	MK208296	MK193828
*C. ribicola*	*Pinus strobus*	Ontario Canada, 1952	NY00267053	MK208298	MK193829
*C. ribicola*	*Pedicularis yezoensis*	Nagano Japan, 1990	HMAS66843	MK208297	MK193830
*C. strobilinum*	*Pinus taeda*	Florida USA, 1982	FLAS-F-53,222	MK208285	MK193823
*Peridermium filamentosum*	*Pinus ponderosa*	Arizona USA, 1957	MICH300081	MK208299	MK193831
*Quasipucciniastrum agrimoniae*	*Agrimonia pilosa*	Beijing China, 1998	HMAS82312	MK208264	MK193835
*Q. agrimoniae*	*A. pilosa*	Gansu China, 1992	HMAS67301	MK208261	MK193832
*Q. agrimoniae*	*A. pilosa*	Gansu China, 1992	HMAS67302	MK208273	MK193844
*Q. agrimoniae*	*A. pilosa*	Gansu China, 1992	HMAS67306	MK208262	MK193833
*Q. agrimoniae*	*A. pilosa*	Gansu China, 1992	HMAS67309	MK208263	MK193834
*Q. agrimoniae*	*A. pilosa*	Gansu China, 2003	HMAS134791	MK208268	MK193839
*Q. agrimoniae*	*A. pilosa*	Guangxi China, 1997	HMAS77430	MK208271	MK193842
*Q. agrimoniae*	*A. pilosa*	Guizhou China, 2015	HMAS248096	MK208283	MK193854
*Q. agrimoniae*	*A. pilosa*	Heilongjiang China, 2003	HMAS89584	MK208277	MK193848
*Q. agrimoniae*	*A. pilosa*	Heilongjiang China, 2004	HMAS136005	MK208274	MK193845
*Q. agrimoniae*	*A. pilosa*	Heilongjiang China, 2015	HMAS248094	MK208280	MK193851
*Q. agrimoniae*	*A. pilosa*	Heilongjiang China, 2015	HMAS248095	MK208281	MK193852
*Q. agrimoniae*	*A. pilosa*	Inner Mongolia China, 2000	HMAS172175	MK208272	MK193843
*Q. agrimoniae*	*A. pilosa*	Ningxia China, 2000	HMAS172172	MK208270	MK193841
*Q. agrimoniae*	*A. pilosa*	Ningxia China, 2000	HMAS172173	MK208265	MK193836
*Q. agrimoniae*	*A. pilosa*	Sichuan China, 2016	HMAS248093	MK208279	MK193850
*Q. agrimoniae*	*A. pilosa*	Sichuan China, 1989	HMAS63888	MK208266	MK193837
*Q. agrimoniae*	*A. pilosa*	Sichuan China, 1989	HMAS63892	MK208275	MK193846
*Q. agrimoniae*	*A. pilosa*	Sichuan China, 2010	HMAS243033	MK208276	MK193847
*Q. agrimoniae*	*A. pilosa*	Yunnan China, 2007	HMAS199430	MK208269	MK193840
*Q. agrimoniae*	*A. pilosa*	Yunnan China, 2011	HMAS248097	MK208282	MK193853
*Q. agrimoniae*	*A. pilosa*	Tibet China, 2011	HMAAS244481	MK208267	MK193838
*Q. agrimoniae*	*A. pilosa*	Yunnan China, 2016	HMAS248092	MK208278	MK193849

^a^FLAS-F: University of Florida Herbarium, USA; HMAS: Fungarium, Chinese Academy of Sciences, China; MICH: University of Michigan Herbarium, USA; NY: New York Botanical Garden, USA.

**Table 2.** Sequence data retrieved from GenBank and used for phylogenetic analyses.

		**GenBank accession no.**		
**Species**	**Host plants**	**ITS**	**LSU**	**Reference**
*Chrysomyxa arctostaphyli*	*Arctostaphylos sp.*	DQ200930.1	AY700192.1	Matheny et al. (2007)
*C. chiogenis*	*Gaultheria hispidula*	GU049452.1	GU049532.1	Feau et al. (2011)
*C. ledi*	*Picea abies*	HM037711.1	HM037707.1	Kaitera et al. (2010)
*C. ledicola*	*P. mariana*	GU049417.1	GU049520.1	Feau et al. (2011)
*C. nagodhii*	*Rhododendron groenlandicum*	GU049431.1	GU049524.1	Feau et al. (2011)
*C. neoglandulosi*	*Ledum glandulosum*	GU049498.1	GU049550.1	Feau et al. (2011)
*C. piperiana*	*L. macrophyllum*	GU049497.1	GU049565.1	Feau et al. (2011)
*C. rhododendri*	*L. lapponicum*	GU049467.1	GU049560.1	Feau et al. (2011)
*C. rhododendri*	*Rhododendron ferrugineum*	GU049471.1	GU049570.1	Feau et al. (2011)
*C. vaccinii*	*Vaccinium parvifolium*	GU049463.1	GU049561.1	Feau et al. (2011)
*C. woroninii*	*Ledum groenlandicum*	GU049462.1	GU049540.1	Feau et al. (2011)
*Coleosporium. cacaliae*	*Adenostyles alliariae*	KY810462.1	KY810462.1	Beenken et al. (2017)
*Col. campanulae*	*Campanula rapunculoides*	KY810465.1	KY810465.1	Beenken et al. (2017)
*Col. euphrasiae*	*Rhinanthus alectorolophus*	KY810469.1	KY810469.1	Beenken et al. (2017)
*Col. inulae*	*Inula salicina*	KY810470.1	KY810470.1	Beenken et al. (2017)
*Col. ipomoeae*	*Ipomoea sp*	MF769624.1	MF769639.1	McTaggart and Aime (2018)
*Col. petasitidis*	*Petasites hybridus*	KY810471.1	KY810471.1	Beenken et al. (2017)
*Col. plumeriae*	*Plumeria sp*	MF769629.1	GU145555.1	McTaggart and Aime (2018) Holcomb and Aime (2010)
*Col. senecionis*	*Jacobaea alpina*	KY810472.1	KY810472.1	Beenken et al. (2017)
*Col. senecionis*	*Senecio ovatus*	KY810473.1	KY810473.1	Beenken et al. (2017)
*Col. solidaginis*	*Solidago virgaurea*	KY810481.1	KY810481.1	Beenken et al. (2017)
*Col. solidaginis*	*S. gigantea*	KY810483.1	KY810483.1	Beenken et al. (2017)
*Col. tussilaginis*	*Tussilago farfara*	KY810485.1	KY810485.1	Beenken et al. (2017)
*Cronartium conigenum*	*Pinus leiophylla*	L76486.1	*–*	Vogler and Bruns (1998)
*Cro. ribicola*	*–*	DQ533975.1	AF522166.1	–
*Endocronartium harknessii*	*Pinus* sp.	DQ206982.1	AY700193.1	Matheny et al. (2007)
*Hyalopsora polypodii*	*Cystopteris fragilis*	*–*	AF426229.1	Maier et al. (2003)
*H. polypodii*	*Deparia petersenii*	*–*	KJ698627.1	*–*
*Melampsorella caryophyllacearum*	*Caryophyllaceae Cerastium*	*–*	MG907233.1	Aime et al. (2018)
*M. caryophyllacearum*	*Abies alba*	*–*	AF426232.1	Maier et al. (2003)
*M. betulinum*	*Betula pubescens*	KF031556.1	KF031539.1	McKenzie et al. (2013)
*M. betulinum*	*Alnus cordata*	KF031559.1	KF031544.1	McKenzie et al. (2013)
*M. betulinum*	*Betula nana*	KF031562.1	KF031549.1	McKenzie et al. (2013)
*M. hiratsukanum*	*Alnus incana*	KF031554.1	KF031541.1	McKenzie et al. (2013)
*M. hiratsukanum*	*A. rhombifolia*	KC313889.1	KC313888.1	Blomquist et al. (2014)
*Milesina philippinensis*	*Nephrolepis* sp.	*–*	KM249868.1	McTaggart et al. (2014)
*Milesina* sp.	*Pinaceae Abies*	*–*	MG907234.1	Aime et al. (2018)
*M. vogesiaca*	*Polystichum aculeatum*	*–*	MG907235.1	Aime et al. (2018)
*Naohidemyces vaccinii*	*Vaccinium ovatum*	*–*	DQ354563.1	Aime (2006)
*N. vaccinii*	*Vaccinum*	*–*	KJ698628.1	Padamsee et al. (2014)
*Peridermium harknessii*	*Pinus contorta*	L76506.1	*–*	Vogler and Bruns (1998)
*Pileolaria terebinthi*	*Pistacia atlantica*	HM639742.1	HM639742.1	Alaei et al. (2012)
*P. terebinthi*	*P. atlantica*	HM639743.1	HM639743.1	Alaei et al. (2012)
*Pucciniastrum actinidiae*	*Actinidia arguta*	AB221446.1	AB221403.1	Liang (2006)
*P. actinidiae*	*A. rufa*	AB221448.1	AB221405.1	Liang (2006)
*P. boehmeriae*	*Boehmeria tricuspis*	AB221450.1	AB221393.1	Liang (2006)
*P. boehmeriae*	*B. platanifolia*	AB221451.1	AB221391.1	Liang (2006)
*P. corni*	*Cornus kuosa*	AB221437.1	AB221409.1	Liang (2006)
*P. corni*	*C. kuosa*	AB221436.1	AB221408.1	Liang (2006)
*P. fagi*	*Fagus crenata*	AB221425.1	AB221378.1	Liang (2006)
*P. fagi*	*F. crenata*	AB221420.1	AB221374.1	Liang (2006)
*P. fagi*	*F. crenata*	AB221424.1	AB221375.1	Liang (2006)
*P. fagi*	*F. japonica*	AB221421.1	AB221376.1	Liang (2006)
*P. fagi*	*F. crenata*	AB221423.1	AB221377.1	Liang (2006)
*P. hikosanense*	*Acer rufinerve*	AB221441.1	AB221388.1	Liang (2006)
*P. hikosanense*	*A. rufinerve*	AB221440.1	AB221389.1	Liang (2006)
*P. hydrangeae-petiolaris*	*Hydrangea petiolaris*	AB221438.1	AB221384.1	Liang (2006)
*P. hydrangeae-petiolaris*	*H. petiolaris*	AB221439.1	AB221385.1	Liang (2006)
*P. kusanoi*	*Clethra barbinervis*	AB221429.1	AB221400.1	Liang (2006)
*P. kusanoi*	*C. barbinervis*	AB221426.1	AB221402.1	Liang (2006)
*P. kusanoi*	*C.abarbinervis*	AB221430.1	AB221401.1	Liang (2006)
*P. kusanoi*	*C. barbinervis*	AB221427.1	AB221399.1	Liang (2006)
*P. kusanoi*	*Clethra barbinervis*	AB221428.1	AB221398.1	Liang (2006)
*P. miyabeanum*	*Viburnum furcatum*	AB221443.1	AB221397.1	Liang (2006)
*P. miyabeanum*	*V. furcatum*	AB221442.1	AB221394.1	Liang (2006)
*P. styracinum*	*Styrax japonica*	AB221433.1	AB221417.1	Liang (2006)
*P. styracinum*	*S. japonica*	AB221432.1	AB221418.1	Liang (2006)
*P. styracinum*	*S. japonica*	AB221431.1	AB221416.1	Liang (2006)
*P. tiliae*	*Tilia mandshurica*	AB221455.1	AB221412.1	Liang (2006)
*P. tiliae*	*T. japonica*	AB221453.1	AB221414.1	Liang (2006)
*P. tiliae*	*T. japonica*	AB221454.1	AB221415.1	Liang (2006)
*P. yoshinagai*	*Stewartia monadelphagene*	AB221435.1	AB221410.1	Liang (2006)
*P. yoshinagai*	*S. monadelphagene*	AB221434.1	AB221411.1	Liang (2006)
*Quasipucciniastrum agrimoniae*	*Agrimonia eupatoria*	*–*	AF426234.1	Maier et al. (2003)
*Q. agrimoniae*	*–*	KJ486537.1	KJ725376.1	Yang (2015)
*Q. agrimoniae*	*–*	KJ486536.1	KJ725375.1	Yang (2015)
*Q. agrimoniae*	*Agrimonia* sp.	*–*	MG907236.1	Aime et al. (2018)
*Thekopsora guttata*	*Galium odoratum*	*–*	AF426231.1	Maier et al. (2003)
*T. minima*	*Ericaceae Vaccinium*	*–*	MG907243.1	Aime et al. (2018)
*T. minima*	*Vaccinium corymbosum*	*–*	KY991374.1	Shands et al. (Unpublished)
*T. symphyti*	*Symphytum officinale*	*–*	AF426230.1	Maier et al. (2003)
*Uredinopsis osmundae*	*Dryopteridaceae Athyrium*	*–*	MG907244.1	–
*U. osmundae*	*Osmundaceae Osmunda*	*–*	MG907245.1	–
*U. pteridis*	*Pteridium esculentum*	*–*	KM249869.1	McTaggart et al. (2014)
*Uredinopsis* sp.	*–*	*–*	AF522181.1	–

(–): No information from GenBank.

Sequences were manually aligned by using BioEdit 7.0.9 (Hall 1999), and multiple alignments were performed using MAFFT 7 (Katoh and Standley 2013). Gaps were treated as missing data for all analyses. The Akaike Information Criteria (AIC) in Modeltest 3.7 (Posada and Crandall 1998) was used to estimate the best-fit substitution models, and GTR+I + G was selected as the best evolutionary model. Maximum Likelihood (ML) analyses were performed using RAxML 8.0.0 (Stamatakis and Alachiotis 2010), and Bayesian Markov chain Monte Carlo (MCMC) analyses were performed by MrBayes 3.1.2 (Huelsenbeck and Ronquist 2001). Supported values of ML and Bayesian posterior probability (Bpp) were indicated in the phylogenetic tree.

## Results

### Molecular phylogeny

**Figure 1.**
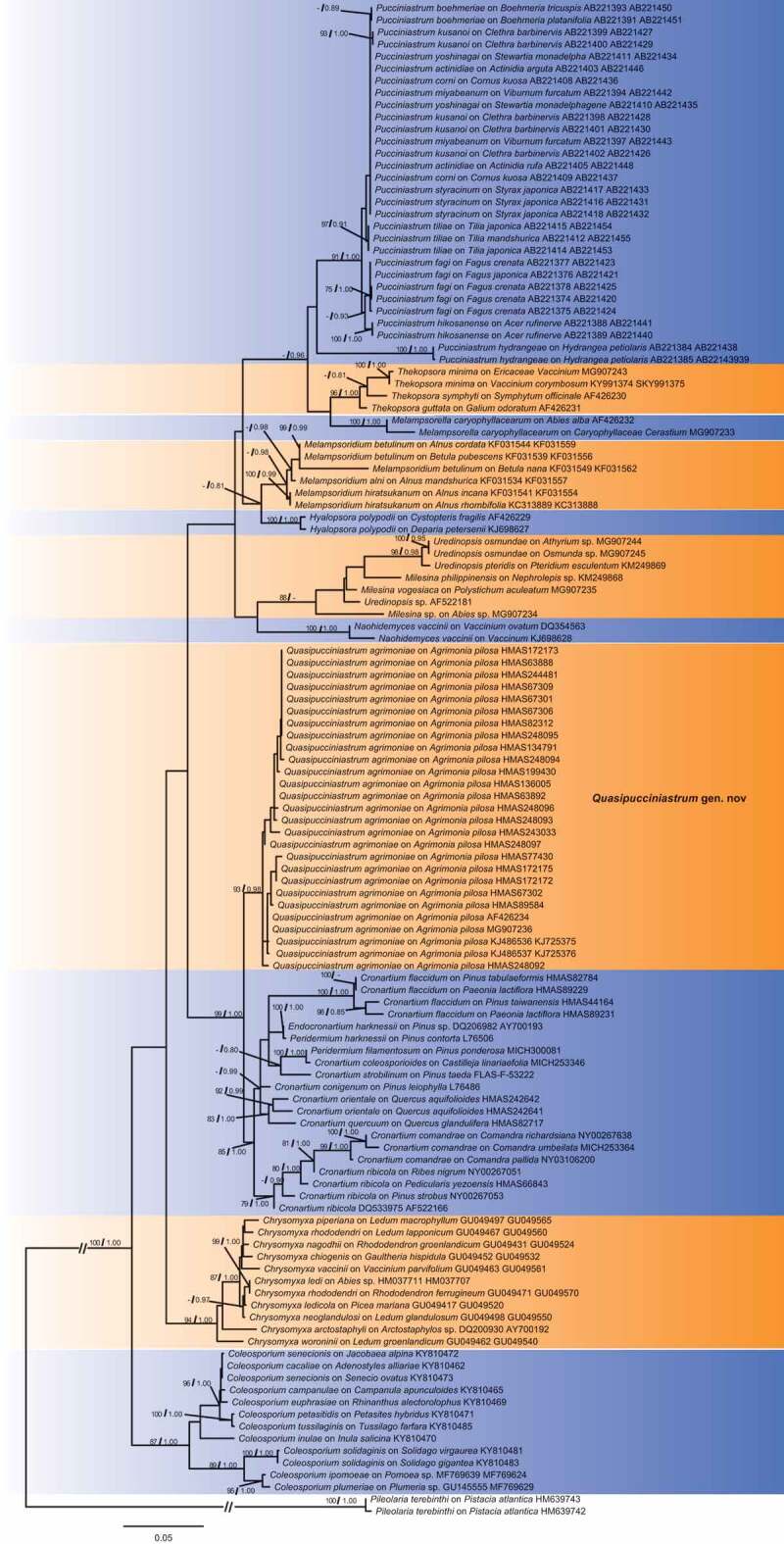
Bayesian 50% majority-rule consensus tree based on concatenated data of rDNA ITS and LSU sequences. *Pileolaria terebinthi* was used as outgroup. Values on the branches indicate maximum likelihood bootstrap values and Bayesian posterior probabilities. Hyphen indicates that bootstrap values were less than 75% and Bayesian posterior probabilities less than 0.80.

The combined ITS and LSU dataset included 106 sequences of ITS and 123 sequence of LSU from 125 rust samples. The dataset comprised aligned length of 1836 characters, of which, 1162 characters are constant, and 640 are variable with 533 parsimony informative sites. Both ML and Bayesian inference resulted in a highly concordant topology (Figure 1). Our specimens on *Agrimonia pilosa* constituted a strongly supported clade (ML/Bpp = 93/0.98), close to *Cronartium, Endocronartium* Y. Hirats. of the family Cronartiaceae. Genera *Hyalopsora* Magnus, *Melampsorella* J. Schröt., *Melampsoridium* Kleb., *Milesia* F.B. White, *Naohidemyces* S. Sato, Katsuya & Y. Hirats., *Pucciniastrum, Thekopsora* Magnus and *Uredinopsis* Magnus from the family Pucciniastraceae, which produce *Milesia*-type uredinia and multicellular teliospores, clustered together but are phylogenetically distant from our rust specimens on *A. pilosa*.

***TaxonomyQuasipucciniastrum*** X.H. Qi, P. Zhao & L. Cai, **gen. nov**.MycoBank no.: MB828669

#### Etymology

*Quasipucciniastrum* (Lat.) referring to the morphological characters (uredinial and telial stages) similar to genus *Puccinistrum*.

#### Type species

*Quasipucciniastrum agrimoniae* X.H. Qi, P. Zhao & L. Cai

#### Generic diagnosis

Spermogonia and aecia unknown. Uredinia hypophyllous, subepidermal, round, scattered or crowded in groups, *Milesia*-type, erumpent with peridium opening by a pore delimited by well-developed, globose and verrucose ostiolar cells, peridial cells small, irregular. Urediniospores borne singly, no pedicels, ovoid, globoid or ellipsoid, spore wall colourless, echinulate. Telia hypophyllous, subepidermal, one spore deep, light yellow. Teliospores subglobose, sessile, multicellular, separated by vertical or oblique septa, wall apparently thickened at apex.

#### Notes

The new genus *Quasipucciniastrum* is characterised by its *Milesia*-type uredinia with well-developed ostiolar cells, well-developed peridial cells, hypophyllous telia producing subglobose teliospores which is divided by vertical and oblique septa under host epidermis. This genus resembles *Pucciniastrum* (Pucciniastraceae, Pucciniales) but differs in producing hypophyllous telia, subglobose teliospores with apparently thickened apical wall. Within family Pucciniastraceae, other genera clearly differed from *Quasipucciniastrum* in uredinial ostiole and teliospores. Genera *Calyptospora, Hyalopsora, Milesina* and *Uredinopsis* differ from this new genus in the type of ostiole, position of telia and type of teliospores, while *Melampsorella* and *Melampsoridium* differ from *Quasipucciniastrum* mainly in their unicellular teliospores without septa (Cummins and Hiratsuka 2003). In addition, *Melampsorella* has *Milesia*-type uredinia with discrete ostiole, which also clearly differs from *Quasipucciniastrum*. Phylogenetic results supported the separation of *Quasipucciniastrum* from *Pucciniastrum, Melampsorella* and other genera in the family Pucciniastraceae (Figure 1).

**Figure 2.**
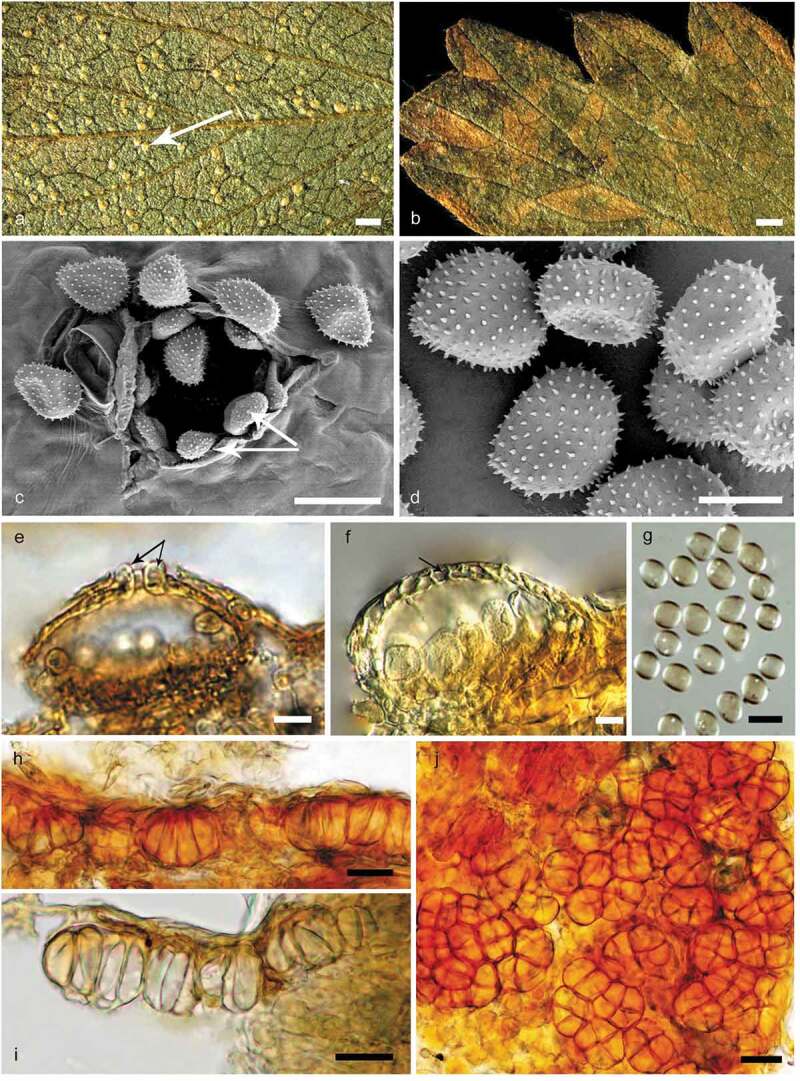
Morphological characters of *Quasipucciniastrum agrimoniae*. A: Uredinia (white arrow) on the hypophyllous leaf surface. B: No uredinia and telia on eiphyllous leaf surface. C: Uredinium with echinulate ostiolar cells (white arrow) observed by SEM. D: Urediniospores with echinulate spines observed by SEM. E: Uredinium with well-developed ostiolar cells (black arrow) observed by LM. F: Uredinium with peridium cell (black arrow). G: Urediniospores observed by LM. H: Vertical section of hypophyllous telia. I: Subepidermal teliospores with apparently thickened apical wall. J: Subglobose teliospores with vertical or oblique septa. Bars: A, B = 0.6 mm; C, E, G, H, I, J = 20µm; D, F = 10µm.

***Quasipucciniastrum agrimoniae*** X.H. Qi, P. Zhao & L. Cai, **sp. nov. (**Figure 2)MycoBank no.: MB828670

#### Typification

CHINA. HEILONGJIANG: Greater Higgnan Mountains, Tahe, II, III on *A. pilosa*, 6 September 2015, P. Zhao (**Holotype designated here**, HMAS248095), GenBank: ITS = MK208281; LSU = MK193852.

#### Etymology

Named after the host plant of the type specimen.

#### Description from holotype

Spermogonia and aecia unknown. Uredinia hypophyllous, subepidermal, round, pale-yellow, scattered or rarely grouped, 0.1–0.4 mm in diameter, erumpent with peridium with well-developed ostiolar cells, usually six to nine, arranged at the apex of uredinia. Peridial cells small, irregular, walls smooth. Ostiolar cells well-developed, oblong or ellipsoid, with rough surface. Urediniospores borne singly, no pedicels, scattered, globoid, ovoid or ellipsoid, 16.5–22 × 12.5–17.3 µm, wall 0.7–2.1 µm thick, evenly echinulate without smooth area on surface, spinules 0.5–1.2 µm in length, and the distance between spinules 1.1 and 2.2 µm, germ bizonated. Telia hypophyllous, subepidermal, one spore deep, light yellow. Teliospores consisting of several cells adhering laterally under the epidermal cells, sessile, subglobose, 18.5–36.1 × 13.7–29.9 µm, 2–6 celled, mainly with vertical septa, walls apparently thickened at apex, up to 4.6 µm.

Additional specimens examined: CHINA. BEIJING: Dongling Mountain, II, III on *Agrimonia pilosa*, 16 September 1998, J.Y. Zhuang (HMAS82312); CHINA. GANSU: Gan Nan, Diebu, II, III on *A. pilosa*, 10 September 1992, J.Y. Zhuang (HMAS67301); CHINA. GANSU: Gan Nan, Diebu, II, III on *A. pilosa*, 12 September 1992, J.Y. Zhuang (HMAS67302); CHINA. GANSU: Gan Nan, Zhouqu, II, III on *A. pilosa*, 5 September 1992, J.Y. Zhuang (HMAS67306); CHINA. GANSU: Lan Zhou, Yongdeng, II, III on *A. pilosa*, 11 October 2003, J.Y. Zhuang (HMAS134791); CHINA. GANSU: Long Nan, Wenxian, II, III on *A. pilosa*, 21 September 1992, J.Y. Zhuang (HMAS67309); CHINA. GUIZHOU: Qian Nan, Maolan, II, III on *A. pilosa*, 21 June 2015, P. Zhao (HMAS248096); CHINA. HEILONGJIANG: Greater Higgnan Mountains, Jiagedaqi District, II, III on *A. pilosa*, 9 September 2015, P. Zhao (HMAS248094); CHINA. HEILONGJIANG: Khakan Nature Reserve, II, III on *A. pilosa*, 9 August 2003, J.Y. Zhuang (HMAS89584); CHINA. HEILONGJIANG: Mu Dan Jiang, II, III on *A. pilosa*, 9 August 2004, J.Y. Zhuang (HMAS136005); CHINA. INNER MONGOLIA: Tong Liao, Horqin, II, III on *A. pilosa*, 18 September 2000, J.Y. Zhuang (HMAS172175); CHINA. NINGXIA: Jin Yuan, II, III on *A. pilosa*, 31 August 2000, J.Y. Zhuang (HMAS172172); CHINA. NINGXIA: Jin Yuan, II, III on *A. pilosa*, 1 September 2000, J.Y. Zhuang (HMAS172173); CHINA. SICHUAN: Liangshan Yi Autonomous Prefecture, Meigu, II, III on *A. pilosa*, 8 October 1989, J.Y. Zhuang (HMAS63892); CHINA. SICHUAN: Liangshan Yi Autonomous Prefecture, Yanyuan, II, III on *A. pilosa*, 11 September 2010, J.Y. Zhuang (HMAS243033); CHINA. SICHUAN: Tibetan Qiang Autonomous Prefecture of Ngawa, Wolong, II, III on *A. pilosa*, 23 September 1989, J.Y. Zhuang & S.X. Wei (HMAS63888); CHINA. SICHUAN: Yi Bin, Xingwen, II, III on *A. pilosa*, 21 June 2016, P. Zhao (HMAS248093); CHINA. TIBET: Rikaze, Yadong, II, III on *A. pilosa*, 20 August 2011, J.Y. Zhuang & T.Z. Wei (HMAS244481); CHINA. YUNNAN: Hong He, Binbian, II, III on *A. pilosa*, 19 September 2007, J.Y. Zhuang (HMAS199430); CHINA. YUNNAN: Hong He, Mengzi, II, III on *A. pilosa*, 14 June 2016, P. Zhao (HMAS248092); CHINA. YUNNAN: Kun Ming, II, III on *A. pilosa*, 17 June 2016, P. Zhao (HMAS248097).

Hosts of uredinial and telial stages and geographical distribution: *Agrimonia pilosa* – China: Beijing, Gansu, Guangxi, Guizhou, Heilongjiang, Inner Mongolia, Ningxia, Sichuan, Tibet, Yunnan.

## Discussion

In this study, we recognised a new genus *Quasipucciniastrum* on *Agrimonia pilosa*, and described a new species *Q. agrimoniae* based on morphological and molecular evidences. Hitherto, rust species in genera *Puccinia* Pers., *Pucciniastrum, Thekopsora, Uredo* Pers. and *Uropyxis* J. Schröt. have been recorded on *Agrimonia* species, but *Quasipucciniastrum* clearly differs from above-mentioned genera by its hypophyllous telia, subepidermal teliospores with subglobose shape, and multicellular teliospores with thickened apical wall. rDNA based phylogenies further supported the independence of *Quasipucciniastrum* from these genera, especially *Pucciniastrum* and other genera in Pucciniastraceae, which have similar uredinial and telial morphologies. Here we confirmed the close relationship of *Quasipucciniastrum* and *Cronartium* (Figure 1). *Quasipucciniastrum* is currently best placed in Cronartiaceae, together with *Cronartium*, although this should be better confirmed after the examination of the morphological characters in spermogonial and aecial stages of *Q. agrimoniae*. Morphologically, genus *Cronartium* owned *Milesia*-type uredinia that is similar to *Quasipucciniastrum*, but its columnar telia and unicellular teliopsores are embedded in a common matrix (Cummins and Hiratsuka 2003). Hitherto, we are not successful to obtain the spermogonial and aecial stages of *Q. agrimoniae*. Further investigation on its life cycles and detailed morphological examination of spermogonia and aecia are necessary.

The rust fungus on *Agrimonia pilosa* was previously frequently recognised as *Pucciniastrum agrimoniae* due to its ostiolar cells and subepidermal teliospores divided by vertical septa (Tai 1979; Guo 1989; Zhang et al. 1997; Cao and Li 1999; Zhuang and Wei 1999; Cao et al. 2000; Zhuang 2001, 2005). *P. agrimoniae* was first described on *A. pilosa* from Western Siberia Borus Mountains, Altai in Russia by Tranzschel (1895). Based on the original description of *P. agrimoniae* from its type specimens and other Russian materials (Tranzschel 1895; Sydow and Sydow 1915; Kuprevich and Tranzschel 1957), our novel species *Quasipucciniastrum agrimoniae* on *A. pilosa* resembles *P. agrimoniae* in several aspects but still clearly differs in its 2 to 6 celled and subglobose teliospores with apparently thickened apical wall. Similarly *P. agrimoniae-*like rust on *A. eupatoria* was once reported to bear phylogenetic affinities to *Cronartium flaccidum* and *C. ribicola* (Cronartiaceae) Maier et al. (2003). Those specimens used by Maier et al. (2003) have been shown to be conspecific to *Q. agrimoniae* in our study. As for *P. agrimoniae*, currently there is no type-derived sequence to confirm its phylogenetic affinities. Since the type specimen of *P. agrimoniae* was not obtained from all possibly deposited herbaria, it might have been lost. Neotypification is thus needed using a new and suitable specimen from the original host and locality.

Hitherto, several genera in Pucciniastraceae have been delimitated based on morphological characters in teliospores (Cummins and Hiratsuka 2003), even at generic level, the position of telia and morphology of teliospores have long been used as important taxonomic criteria (Hiratsuka 1958; Cummins and Hiratsuka 2003; Liang et al. 2005; Yang 2015). Although there have been much debates concerning the generic classification based on telial morphologies, recent phylogenetic studies and our study supported the monophyly of all sampled genera in Pucciniastraceae, thus, supported the effectiveness of telial morphologies as taxonomic criteria at generic level. In addition, our morphological and molecular studies further emphasised the importance of the uredinial morphology at generic level because *Quasipucciniastrum* and *Cronartium* with *Milesia*-type uredinia show a much closer relationship than those with *Caeoma*-type uredinia (*e.g*. *Cronartium, Chrysomyxa*). These overlooked morphological characters appeared to be very useful in delimiting taxa at generic and suprageneric level. Further comprehensive studies need to be conducted to evaluate the effectiveness of these morphological characters in rust taxonomy.
